# Research on the Effect of MT+FES Training on Sensorimotor Cortex

**DOI:** 10.1155/2022/6385755

**Published:** 2022-06-03

**Authors:** Yuan-xing Wang, Zhi-zeng Luo

**Affiliations:** Institute of Intelligent Control and Robotics, Hangzhou Dianzi University, Hangzhou 310018, China

## Abstract

**Purpose:**

Aiming at the motor recovery of patients with unilateral upper limb motor dysfunction after stroke, we propose a mirror therapy (MT) training method, which uses surface electromyography (sEMG) to identify movements on one side and control the other side to perform functional electrical stimulation (FES) while mirror therapy is used. And we verify the effect of this training method by analyzing the activity changes of the sensorimotor cortex.

**Method:**

Ten subjects (6 men and 4 women) were randomly divided into two groups according to 3 men and 2 women in each group: the experimental group (*n* = 5) received FES+MT training, and the control group (*n* = 5) received MT training. Both groups were trained at a fixed time at 9 : 00 am every day, each time lasting 20 minutes, once a day, 5 days a week, continuous training for 4 weeks, and the training action was elbow flexion training. During the training of the elbow flexion exercise, the experimental group applied FES with a frequency of 30 Hz, a pulse width of 100 *μs*, and a current of 10 mA to the muscles corresponding to the elbow flexion exercise, and rested for 10 s after 10-s stimulation. We collect the EEG of the elbow flexion motor imagery of all subjects before and after training, and calculate the eigenvalue *E*, and analyze the effect of FES+MT training on the activity of the cerebral sensorimotor cortex.

**Results:**

After repeated measure (RM) two-way ANOVA of the two groups, comparing the subjects' *μ* rhythm elbow flexion motor imagery eigenvalue E, the experimental group (after training) > the control group (after training) > before training.

**Conclusion:**

The FES+MT training method has obvious activation effect on the cerebral sensorimotor cortex.

## 1. Introduction

It is estimated that 4.5 million people die from stroke each year in the world. By 2023, the number of first stroke patients will increase by about 30% compared with 1983 [1]. After the onset of a stroke, it may cause damage to the sensorimotor system, causing 50% of survivors to have hemiplegia and upper limb injuries [2]. The theory of neuroplasticity points out that timely and reasonable autonomous rehabilitation of the damaged nervous system can rebuild the motor nerve pathway and restore motor function to a certain extent [3]. The motor dysfunction caused by stroke comes from the motor cortex, so repairing the function of the motor cortex is the fundamental goal of rehabilitation for stroke patients with motor dysfunction.

A commonly used and effective rehabilitation method is functional electrical stimulation (FES) [4], which externally stimulates the patient's motor-impaired limb muscles through a preset current pulse sequence to improve the nerve conduction function of the corresponding muscle group [5], and through the sensory pathway, the proprioception generated by FES intervention is transferred to the cerebral cortex to promote the remodeling of the sensorimotor cortex [6]. Previous studies [7] performed FES stimulation for stroke patients with upper limb dysfunction twice a day for 30 minutes each for six weeks to produce repetitive wrist extensions, which improved the strength and grip strength of the wrist extensors. And it also proved that FES can promote the rehabilitation of patients' wrist function. Another common rehabilitation training method is mirror therapy (MT). As a common rehabilitation therapy, MT is usually done by placing a mirror between the patient's arms or legs, and through the movement of the uninhibited limb, motor imagery of the affected limb is produced [8–10]. A study [9] through a comparative experiment between mirror therapy and traditional rehabilitation plans found that mirror therapy has a better rehabilitation effect and can stimulate the remodeling of the sensorimotor cortex of the patient's brain. There is also a study [10] using standardized mean difference, mean difference, and odds ratio parameters to conduct rating analysis on the results of randomized controlled trials of multiple groups of mirror therapy and other therapies, confirming that mirror therapy is effective in improving upper limb motor function and activities of daily living. In addition to the above two methods, motor imagery can activate the motor-related cerebral cortex like actual sports [11], and promote the reorganization or reconstruction of the sensorimotor cortex function, so it is also used for active rehabilitation training for patients with stroke motor dysfunction [12]. For decades, electroencephalography (EEG) has been used to study stroke [13]. And as early as 1999, a study [14] has confirmed that when preparing and performing an action with one hand, the contralateral sensory cortex EEG signal has a decrease in the amplitude of *α* rhythm (8-12 Hz) and *β* rhythm (13-30 Hz), and said it is called event-related desynchronization (ERD); at the same time, the amplitude of *α* rhythm and *β* rhythm in the cerebral cortex signal on the same side of the moving hand increases, which is called event-related synchronization (ERS). After the researchers further divided the frequency bands, the alpha rhythm in the central area of the brain is also called the *μ* rhythm. The *μ* rhythm mainly appears in the motor sensory area, and the change rate of the ERD energy of the *μ* rhythm can be used to detect the activity of the motor cortex [15].

Based on the above theories, with the purpose of improving the activity of the cerebral sensorimotor cortex and promoting the remodeling of the cortex, we propose a training method combining FES+MT. The specific steps of this method are as follows: The subject performs the same motion task on both sides at the same time, and only performs motor imagination on one side, and does not perform actions. At the same time, collect the surface electromyography signal (sEMG) of the other side to perform the action. After the action is recognized, the FES is applied to the muscle group corresponding to the action performed on the motor imagery side through the recognized action control. We assume that this training method can increase the activity of the cerebral sensorimotor cortex and mark the subjects trained by this method as the experimental group. We collected the EEG of the subject's right-hand elbow flexion motor imagery before and after training and calculated the eigenvalue *E* constructed by the change rate of the ERD energy of the *μ* rhythm between the left and right central regions of the brain during the motor imagery and compared the results with those of the control group, only MT training without FES, and analyzed the effect of the combined training method of FES+MT on the activity of the sensorimotor cortex.

## 2. Methods

### 2.1. Participants

We recruited 10 healthy subjects (6 males and 4 females), aged 23 ± 2 years old, all right-handed, in good physical condition, and equivalent in education. We arranged 10 subjects for MT+FES training and MT training, respectively. During training, subjects performed training movements on the right side and only motor imagery on the left side (to simulate patients with left-sided dyskinesia). We randomly divided 10 subjects (6 males and 4 females) into an experimental group (MT+FES) and a control group (MT), with 3 males and 2 females in each group, and randomly numbered the subjects in the experimental group (1-5), and in the control group, subjects were randomly numbered (6-10) and compared the effects of two training methods on the activity of the cerebral sensorimotor cortex.

### 2.2. Procedure

This experiment adopts a randomized controlled experiment design and is divided into experimental group (*n* = 5) and control group (*n* = 5). After the experiment started, the two groups of subjects received elbow flexion training at a fixed time at 9 : 00 am every day, each time lasting 20 minutes, once a day, training 5 days a week, continuous training for 4 weeks. After hearing the cue to start the training, the subjects imagined that both arms were doing elbow flexion at the same time, while the right side was performing elbow flexion. In the control group, subjects enhanced their imagination of the left elbow flexion by looking at the movements in the mirror. And in the experimental group, the subjects not only observed the movements through the mirror, but also identified the right movements through EMG during the execution of the right movements (the method of sEMG recognition adopts the method of EMG signal gesture recognition based on convolutional network mentioned in study [16]) and controlled the corresponding muscles of the left arm to apply FES stimulation. The specific identification and stimulation operation were as follows: When subjects in the experimental group performed the elbow flexion movement, a disposable FES electrode patch was attached to the muscle corresponding to the elbow joint flexion movement. When elbow flexion is performed on the right side, after sEMG recognition, the FES instrument synchronously applies FES with a frequency of 30 Hz, a pulse width of 100 *μ*s, and a current of 10 mA to the corresponding muscles on the left side. During training, we also ensured that subjects performed 10 seconds of stimulation and 10 seconds of rest training movements. And after sEMG recognition of the action, the time interval between FES to stimulate the other side of the muscle is controlled within 100 ms to ensure synchronization.  Table 1 summarizes the upper limb muscles and their functions corresponding to the target exercise.

**Figure 1:**
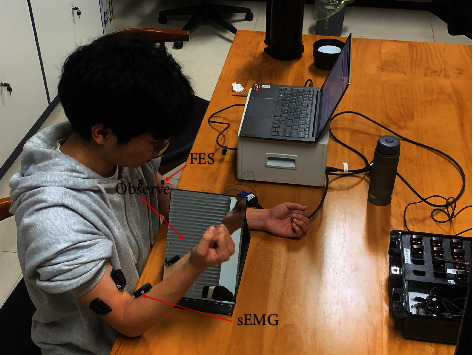
A subject in the experimental group undergoing rehabilitation training.

When performing other exercises (such as wrist flexion) training, we can change the position of the FES electrode patch according to the corresponding muscles shown in Table 1. And Figure 1 shows the specific situation of a subject in the experimental group during training. In Figure 1, the computer sends out the elbow flexion training instruction; the subject imagines the arms to do the elbow flexion training according to the instruction. After the EMG signal is collected by EMG acquisition device on the right side, it is transmitted to the computer through Bluetooth. And the computer controls the functional electrical stimulator to apply FES to the left muscle according to the recognition results (in this experiment, the training action is elbow flexion, so only the biceps and triceps are stimulated).

### 2.3. Data Collection

**Figure 2:**
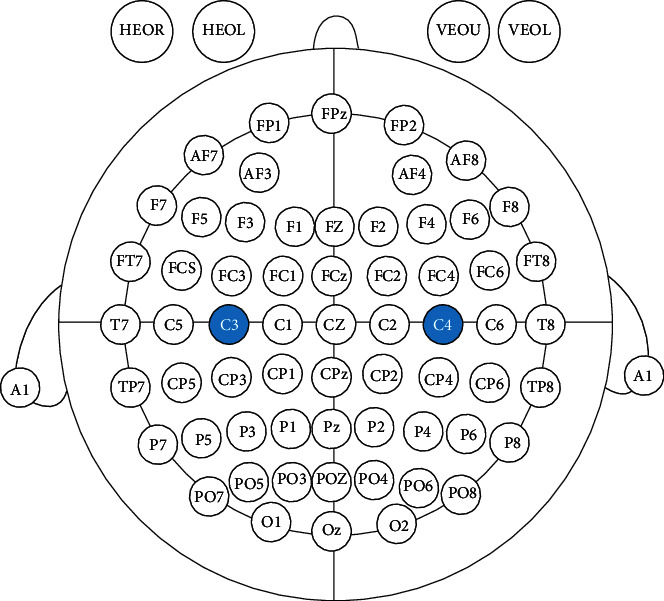
Location map of EEG collection.

For the selection of EEG acquisition channels, we refer to reference [17] and select the left central area (C3) and right central area (C4) related to the motor imagery of the arms, and the time-frequency maps obtained were used for selection of the mu rhythms with the most significant band power increase or decrease during the motor imagery tasks at the central electrode positions C3 and C4. We collect the EEG before and after the training of the subjects, and calculate the eigenvalue *E* constructed by the ERD energy change rate of the *μ* rhythm, which is used as an indicator of the activity of the cerebral cortex. Therefore, we collected the EEG of the right upper limb motor imagery before the elbow flexion training in the experimental group and the control group and the EEG of the left upper limb motor imagery after completing a cycle of training. The EEG collection before and after this training uses the 64-lead EEG collection instrument of the Neuracle Company, and the electrodes are in accordance with the international standard 10-20 lead. During the motor imagery process, the right central area (C4) of the contralateral brain area corresponding to the left upper limb showed ERD due to the motor imagery of the left hand, while the left central area (C3) on the same side did not show ERD. Therefore, we only collect the EEG of C3 and C4 for this experiment. During the collection process, the reference electrode REF is connected, the GND electrode is grounded, and the sampling frequency is 1000 Hz. The notch filter is used during the collection to eliminate 50 Hz power frequency interference.  Figure 2 shows the location of EEG collection.

**Figure 3:**
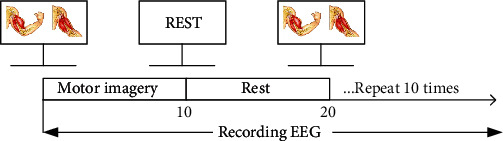
Experimental paradigm sequence diagram.

The specific method of EEG collection for the subjects in the experimental group and the control group is as follows: the subjects sit in the middle of the chair and guide the subjects' left arm to do elbow flexion motor imagery through the screen pictures, rest for 10 seconds after the motor imagery, and repeat 10 times.  Figure 3 is the sequence diagram of EEG collection.

### 2.4. Measurement

We collected the EEG of C3 and C4 leads during elbow flexion motor imagery of the left arm and calculated the energy change rate of ERD before (resting state) and after elbow flexion motion imagination in *μ* rhythm as an indicator of the activity degree of the cerebral sensorimotor cortex. And we also define the absolute value of the difference between the ERD energy change rate in leads C3 and C4 as *E*. The calculation steps are as follows [18]:

Step 1, data preprocessing, we cannot avoid blinking during the collection of elbow flexion's motor imagery EEG. The electrooculogram (EOG) signals caused by eyeball or eyelid movement propagates along the skull and merges with the EEG signals, causing the EEG to produce artifacts. EOG artifacts are one of the main noises in EEG signals. After 50 Hz power frequency filtering, we use wavelet threshold method to remove high frequency noise and then use fast independent component analysis (FastICA) algorithm to remove EOG artifacts. After that, we perform 8 ~ 12 Hz band-pass filtering on the EEG collected in leads C3 and C4.

Step 2, calculate the mean value of all EEG sampled values, and calculate the mean value of the difference squared.

(1) $$\begin{array}{c} A_{j}=\frac{1}{N-1}\overset{N}{\underset{i=1}{\sum }}{\left(x_{ij}-\bar{x_{j}}\right)}^{2}. \end{array}$$

In formula (1), *x*_*ij*_ is the value of the *j*^*th*^ sample during the *i*^*th*^ data collection, $\bar{x_{j}}$ is the average value of all the *j*^*th*^ sampling points, *N* is the total number of data collection, and *A*_*j*_ is the mean value of the difference squared.

Step 3, calculate ERD energy change rate.

(2) $$\begin{array}{c} R=\frac{1}{s-r}\overset{s}{\underset{j=r}{\sum }}A_{j}, \end{array}$$

(3) $$\begin{array}{c} \text{ERD}=\frac{1}{\text{n}-m}\overset{n}{\underset{j=m}{\sum }}\left(A_{j}-R\right)/R. \end{array}$$

where in formula (2), [*r*, *s*] is the resting interval, and *R* is the mean value of the square of the sample value difference in the resting interval, and in formula (3), [*m*, *n*] is the motor imagery interval, and ERD is the rate of energy change.

Step 4, calculate the eigenvalue *E*.

(4) $$\begin{array}{c} E_{a}=\left|{\text{ERD}}_{a}^{C3}-{\text{ERD}}_{a}^{C4}\right|, \end{array}$$

where *E*_*a*_ is the eigenvalue *E* calculated by th*ea*^*th*^ subject and *ERD*_*a*_^*C*3^(*ERD*_*a*_^*C*4^) is the ERD energy change rate during motor imagery in lead C3 (C4).

**Figure 4:**
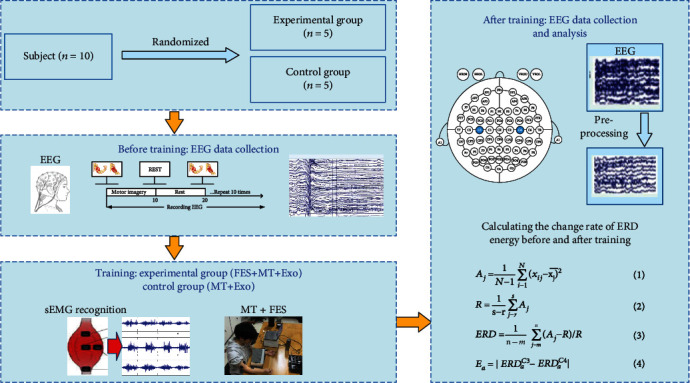
Experimental design flow.

Thus, through the above calculation method, we can obtain the eigenvalue *E* of the two groups of subjects before and after the elbow flexion training. Because the executive action or motor imagery in left side is controlled by the contralateral brain area (C4), when the subject's left arm performs the elbow flexion motor imagery, the ERD phenomenon will occur in lead C4 under the *μ* rhythm, and the amplitude of its frequency spectrum related to elbow flexion is suppressed. At the same time, an ERS phenomenon will occur in the ipsilateral brain area (C3). After elbow flexion training, the sensorimotor cortex becomes active, making the ERD phenomenon of the contralateral brain area more obvious, and the eigenvalue *E* *o*btained according to the formula will increase. Similarly, when the right side is the training side, if the training is effective, the ERD phenomenon of the contralateral brain area (C3) of the right arm will be more obvious, and *E* will also increase. Therefore, in this paper, we are only doing the left training experiment to verify the effect of MT+FES training. And we use eigenvalue *E* to quantitatively analyze the effect of this training method on the activity of the motor sensory cortex and further speculate on the effect of MT+FES training on sensorimotor cortex remodeling in patients with motor dysfunction.  Figure 4 is the overall flow chart of the experiment in this paper.

## 3. Results

We collected the EEG of the elbow flexion motor imagery of the left arm before and after training in the experimental group and the control group and performed data preprocessing according to Step 1. After that, we calculate the preprocessed C3 and C4 lead EEG according to the steps of formulas (1)~(4) and obtain the elbow flexion motor imagery eigenvalue *E* of the experimental group (MT+FES) and the control group (MT) before and after training. We also calculate the mean and standard deviation of the experimental group and the control group E (Table 2).

In Table 2, ${\bar{E}}_{A}$ and ${\bar{E}}_{B}$are the average values of eigenvalues *E* before and after training in the experimental group and the control group, respectively. According to the data in Table 2, before training, subjects in the experimental group ${\bar{E}}_{A}\pm \sigma =0.62\pm 0.03$; after four weeks of elbow flexion training with MT+FES, ${\bar{E}}_{A}\pm \sigma =0.85\pm 0.05$; and before training, subjects in the control group ${\bar{E}}_{B}\pm \sigma =0.64\pm 0.02$; and after four weeks of elbow flexion training, ${\bar{E}}_{B}\pm \sigma$ = 0.74 ± 0.03.

We compared the experimental group and the control group in their respective groups and found that after the two kinds of training, the eigenvalue *E* of elbow flexion motor imagery increased, indicating that the activity of the cerebral sensorimotor cortex was higher than before training. From the comparison between the groups, it is obvious that before training, the experimental group and the control group have no significant difference in the elbow flexion motor imagery eigenvalue *E* of the subjects. After the training, the MT+FES training method improves the motor imagery eigenvalue *E* more significantly than the control group. In order to quantitatively analyze the difference of *E* between the two groups before and after elbow flexion training, we used repeated measure (RM) two-way ANOVA to analyze the data differences between the experimental and control groups before and after training. In this experiment, since the sphericity test result *P* < 0.05, the data does not meet the spherical assumption, so when comparing within groups, the multivariate analysis of variance shall prevail (Table 3). And the between-group factors were tested by one-way ANOVA (Table 4).

Within-group comparison, according to the Pillai's trace results in Table 3, both time and time∗group (training method)'s *P* < 0.05, indicating that as the training progresses, the eigenvalue *E* is significantly affected by the training time. And there is an interaction effect between training time and training method. Between-group comparison, according to Table 4, *P* < 0.05, there is a difference in the influence of the two training methods on the eigenvalue *E*. Based on the above results, we believe that both the MT training method and the FES+MT training method can improve the activity of the sensory motor cortex of the brain and the MT+FES training method is more effective.

## 4. Conclusion and Discussion

In this paper, we studied the effect of the combined training method of FES+MT on the activity of the sensorimotor cortex. The results show that both the experimental group and the control group increased the activity of the subjects' sensory motor cortex after training. Combined with the theory of neuroplasticity [3], we believe that this training method is effective in remodeling the motor cortex of patients with poststroke dyskinesia and can improve the motor function of the upper limbs of patients with unilateral upper limb dyskinesia after stroke.

In terms of rehabilitation of poststroke patients, previous studies have verified the significant effect of FES on improving the upper limb function of stroke patients through Fugl-Meyer evaluation grade [19], muscle strength [20], and other aspects. Based on FES, different training programs have also been designed for the rehabilitation of stroke patients. Meng et al. [21] aimed at the rehabilitation training of upper limb motor function of patients with chronic stroke, through the brain-computer interface (BCI) to control FES for training. After 10 trainings, the error rate of BCI was less than 20%, which proves that the BCI+FES rehabilitation training is feasible. Wang et al. [22] developed a mirrored upper limb rehabilitation robot system, which includes an exoskeleton (Exo) upper limb assisting robot and a healthy side information acquisition system, which uses the healthy side acceleration (ACC) to recognize gestures to drive rehabilitation training on the affected side. Kim et al. [23] studied the effect of FES combined with MT on the upper limb function of stroke patients, and the results showed that FES combined with MT can effectively improve the upper limb motor function. And the above studies confirmed the positive effects of FES and MT on the rehabilitation of motor function of patients after stroke.

Different from the paper [21], we consider the weak recognition of cerebral motor cortex EEG in patients with dyskinesia and the advantages of mirror therapy to improve the motor function of upper limbs in stroke patients. And we propose FES+MT training: FES+MT training is applied to the affected side through the sEMG recognition control of the healthy side. This method is mainly aimed at the rehabilitation of patients with unilateral upper limb motor dysfunction after stroke. For the subjects, the initiative of training at this time is guided by the action of the healthy side. During the training process, bilateral motor imagery and active movement of the contralateral arm make the sensorimotor cortex of the affected side brain stimulated in a mirror therapy manner. After stroke, the sensorimotor cortex of the corresponding brain area of the upper limb with unilateral motor dysfunction has obstacles in motor initiation and cortical coordination. And under the FES+MT training, the muscles corresponding to the movements of the affected side limbs are further stimulated. This stimulation feeds back proprioception to the cerebral sensory cortex through the sensory pathway and positively promotes the remodeling of the sensory motor cortex of the affected side of the brain. As shown in Tables 2, 3, and 4, the FES+MT training method is effective in promoting the activity of the cerebral sensorimotor cortex, and we also found that under elbow flexion training, the FES+MT training method has a better effect on the activation of the cerebral sensorimotor cortex than MT training. Existing clinical rehabilitation training experience shows that unlike healthy people's activity of the cerebral cortex during motor imagination, patients with unilateral motor dysfunction will activate the same side of the brain due to the coordination of the left and right hemispheres of the brain through the corpus callosum, while compensating for the motor function of the contralateral brain area. Previous study [9] has confirmed that when preparing and performing actions with one hand, ERD appears in the sensory cortex of the contralateral brain, and ERS appears in the ipsilateral brain. Therefore, we rationally reason that long-term FES+MT training can promote the normalization of brain function in patients with unilateral motor dysfunction and help remodel the motor cortex nerve.

However, due to the high complexity of the brain and numbers of unknown intercortical coordination functions, our research is still on the surface and speculation. If the training movement is changed to more delicate movements such as wrist flexion or finger flexion, more appropriate cortical activity indicators need to be sought, and whether the increase in cortical activity will promote the correct remodeling of the sensorimotor cortex still needs more clear evidence. And it is also necessary to carry out long-term rehabilitation training for patients and observe the continuous effect.

In summary, in this paper, for patients with unilateral upper limb motor dysfunction after stroke, we propose an MT training method that controls the affected side to perform FES through the contralateral sEMG recognition. And we designed a comparative experiment (control group: MT training) to study the effect of the FES+MT training method on the activity of the patient's sensorimotor cortex. Based on the neural mechanism of ERD/ERS and the generation of *μ* rhythm by the sensorimotor cortex, we choose the absolute value of the difference between the C3 and C4 lead ERD energy change rate of *μ* rhythm as the measure of sensorimotor cortex activity, and collect the EEG of elbow flexion motor imagery on the affected side of the experimental group and the control group before and after training, and calculate the eigenvalue *E* for comparison and analysis. We get the following conclusion: The FES+MT training method has obvious activation effect on the cerebral sensorimotor cortex.

## Figures and Tables

**Table 1:** Target movement corresponds to human's upper limb muscles and their function.

Target movement	Muscle type	Function
Elbow flexion	Biceps brachii	Arm bending and stretching
	Triceps brachii	
Wrist flexion	Extensor carpi radialis	Control wrist abduction
	Flexor carpi radialis	
	Flexor carpi ulnaris	Wrist flexion and adduction
Fingers flexion	Extensor digitorum	Extension of four fingers except thumb
	Extensor pollicis longus	Thumb extensor
	Flexor pollicis longus	Thumb flexion

**Table 2:** The eigenvalue *E* of wrist flexion motor imagery of 10 subjects before and after rehabilitation training.

Group	Subjects	Before training	After training
Experimental	*E*_1_	0.67	0.92
	*E*_2_	0.62	0.86
	*E*_3_	0.61	0.87
	*E*_4_	0.59	0.78
	*E*_5_	0.63	0.82
	${\bar{E}}_{A}\pm \sigma$	0.62 ± 0.03	0.85 ± 0.05
Control	*E*_6_	0.65	0.79
	*E*_7_	0.62	0.74
	*E*_8_	0.66	0.73
	*E*_9_	0.63	0.75
	*E*_10_	0.62	0.71
	${\bar{E}}_{B}\pm \sigma$	0.64 ± 0.02	0.74 ± 0.03

**Table 3:** Multivariate analysis of variance test results for within-group factors^a^.

Effect		Value	*F*	Hypothesis df	Error df	Sig.
Time	Pillai's trace	.973	293.568b	1.000	8.000	.000
	Wilks' lambda	.027	293.568b	1.000	8.000	.000
	Hotelling's trace	36.696	293.568b	1.000	8.000	.000
	Roy's largest root	36.696	293.568b	1.000	8.000	.000
Time∗ group	Pillai's trace	.821	36.642b	1.000	8.000	.000
	Wilks' lambda	.179	36.642b	1.000	8.000	.000
	Hotelling's trace	4.580	36.642b	1.000	8.000	.000
	Roy's largest root	4.580	36.642b	1.000	8.000	.000

^a^Design: intercept + group. Within subjects design: time. ^b^Exact statistic.

**Table 4:** One-way analysis of variance test results for between-group factors.

Source	Type III sum of squares	Df	Mean square	*F*	Sig.
Intercept	10.182	1	10.182	5168.348	.000
Group	.011	1	.011	5.607	.045
Error	.016	8	.002		

Measure: *E*. Transformed variable: average.

## Data Availability

The EEG data of the subjects used to support the findings of this study have not been made available because subjects are unwilling to disclose their own EEG data, and the eigenvalue *E* can be calculated by publishing EEG data by using the formulas in this study.
